# Rutin–chitooligosaccharide complex: Comprehensive evaluation of its anti-inflammatory and analgesic properties *in vitro* and *in vivo*

**DOI:** 10.1515/biol-2022-1021

**Published:** 2025-03-06

**Authors:** Chuanyun Wen, Mei Zhu, Yin Wang, Jinyu Man, Ramesh Priyanka

**Affiliations:** Department of Anesthesiology, Taizhou People’s Hospital, TaiZhou, 225300, China; Department of Prosthodontics, Saveetha Dental College and Hospital, Saveetha Institute of Medical and Technical Sciences (SIMATS), Saveetha University, Chennai, 600 077, Tamil Nadu, India

**Keywords:** rutin-chitooligosaccharide complex, ROS, inflammation, macrophages, zebrafish

## Abstract

This study investigated the potential anti-inflammatory and analgesic effects of the rutin–chitooligosaccharide (R-COS) complex both *in vitro* and *in vivo*. Initially, the cytotoxicity of R-COS was assessed in RAW 264.7 cells using an MTT assay. Subsequently, an inflammatory study was conducted where R-COS demonstrated a significant reduction in various pro-inflammatory factors (nitric oxide [NO], prostaglandin E2, tumor necrosis factor-α, interleukin-6, interleukin-1, inducible nitric oxide synthase [iNOS], and cyclooxygenase [COX-2]) in lipopolysaccharide (LPS)-stimulated RAW 264.7 cells without compromising cell viability. Furthermore, *in vivo* analysis showcased the protective effect of R-COS on zebrafish embryos exposed to inflammatory stress induced by LPS. R-COS exhibited inhibition against pro-inflammatory factors, specifically COX-2 and iNOS. Notably, R-COS played a modulatory role in calcitonin gene-related peptide and NO levels in zebrafish, reducing the expression of pro-inflammatory factors. Additionally, the study observed analgesic activity in zebrafish treated with R-COS, which mitigated pain-like behavior triggered by acetic acid. Overall, these findings highlight the potential of R-COS, derived from chitosan, as a promising anti-inflammatory agent with broad applications in healthcare and tissue engineering.

## Graphical abstract

**Figure:**
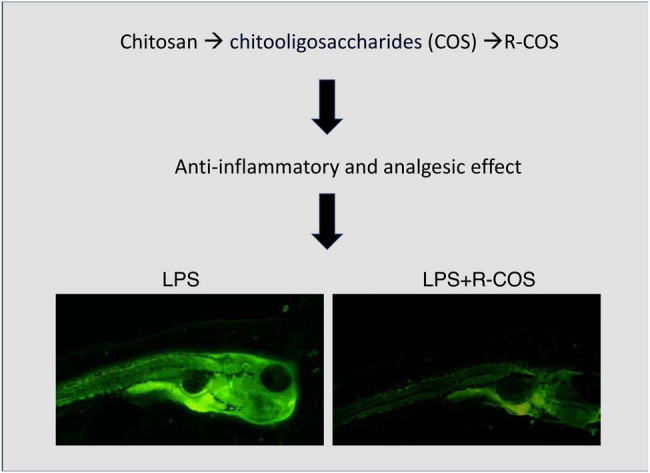

## 1 Introduction

Inflammation is an intricate biological reaction coordinated by the body to safeguard tissues from various stimuli, such as pathogens like bacteria and exposure to chemical agents [1]. Lipopolysaccharide (LPS), also referred to as endotoxin, is commonly employed in scientific research to elucidate the mechanisms involved in inflammatory responses [2]. LPS plays a vital role in activating macrophages by binding to toll-like receptor 4 (TLR4), which then triggers intricate signaling cascades such as MAPK and nuclear factor-kappa B (NF-κB) [3]. The activation of TLR4 by LPS stimulates the synthesis of pro-inflammatory cytokines, including tumor necrosis factor-alpha (TNF-α), nitric oxide (NO), and interleukin-6 (IL-6) [4]. Various investigations have demonstrated that reducing LPS activity can effectively hinder the generation of these inflammatory mediators [5,6,7]. Furthermore, the activation of macrophages also triggers the production of reactive oxygen species (ROS), which could potentially play a role in the development of chronic diseases [8]. The interaction between inflammatory triggers, such as LPS and cellular responses emphasizes the complex network of signaling pathways involved in the inflammatory process and its regulation.

Antioxidants are essential in protecting cells from harm caused by ROS or inflammatory mediators [9]. Chitooligosaccharide (COS) is a water-soluble compound that is obtained from the hydrolysis of chitosan (CS) [10]. COS is present in different sources such as insects and marine organisms. CS, the precursor of COS, demonstrates a wide range of biological activities [10]. Nevertheless, the properties and bioactivity of COS are greatly influenced by the preparation process. The enzymatic approach is frequently utilized for COS synthesis, but it has drawbacks such as being expensive and potentially producing CS with high viscosity, leading to a low level of hydrolysis [10]. The study conducted by Mittal et al. [11] showed that CS (COS) generated utilizing the OH-H_2_O_2_ technique had potent antioxidant and antibacterial characteristics, particularly when 1% hydrogen peroxide (H_2_O_2_) was utilized.

Rutin, which is also known by its chemical name 3,3′,4′,5,7-pentaflavon-3-rhamnoside, is a flavonoid that occurs naturally and can be found in a variety of plants, including citrus fruits and buckwheat flowers. It possesses a wide range of biological actions, such as antioxidant, antibacterial, cholesterol-lowering, and neuroprotective characteristics [6,7,12], among others. The limited solubility and unpleasant taste of rutin significantly impede its effectiveness [7]. Rutin’s effectiveness is significantly hindered. Therefore, it is suggested that COS could potentially interact with rutin, leading to enhanced physical characteristics and bioactivities of rutin as a result of this interaction.

Multiple studies investigating the production of COS have shown that commercially available COS can inhibit the production of pro-inflammatory cytokines IL-6 and TNF-α in LPS-activated RAW264.7 macrophages. Studies by Fernandes et al. [13] and Jitprasertwong et al. [14] demonstrate significant anti-inflammatory benefits in zebrafish models. Although the anti-inflammatory and antioxidant characteristics of COS are acknowledged, there is insufficient evidence regarding the precise mechanism by which rutin–chitooligosaccharide (R-COS) exerts its anti-inflammatory effects and treats oxidative stress [13,14]. Hence, this work aims to examine the impact of different levels of COS on the viability of LPS-activated RAW264.7 cells. The study seeks to comprehensively evaluate the potential of COS in exhibiting analgesic and anti-inflammatory properties using *in vitro* and zebrafish models.

## 2 Materials and methods

### 2.1 COS synthesis

The production of COS was achieved through a sequence of steps using the oxidative hydrolysis method described by Mittal et al. [11]. First, a 1% CS solution was dissolved in a 2% acetic acid (AA) solution and left to dissolve overnight. The pH of this solution was subsequently modified to 5.5 to establish the optimal conditions for the subsequent reaction. The CS solution was treated with H_2_O_2_) and then heated to 60°C. It was kept at this temperature for 2 h to aid the oxidative hydrolysis process. After the reaction, the mixtures were quickly cooled in an ice bath to stop any additional chemical activity, and the pH was adjusted to a neutral value of 7. Afterward, to remove any remaining solid particles that had not dissolved the solution was subjected to centrifugation, which facilitated the separation of the liquid portion, known as the supernatant, that contained the COS. The COS solution was further exposed to lyophilization, a process that involves freeze-drying, in order to remove water and obtain COS particles. Subsequently, the COS powders were meticulously stored at a frigid temperature of −40°C until they were employed for subsequent experiments or analyses, guaranteeing their durability and completeness for future utilization.

### 2.2 R–COS complex

Free rutin combined with COS at a mole ratio of 1:5 was dissolved in water. The solutions were vortexed for 10 min and then centrifuged at 10,000 × *g* at 20°C for 10 min. The resulting supernatant was used for the preparation of spray-dried samples. For spray-drying, the supernatant solutions were subjected to spray-drying at an input temperature of 100°C and a drying air flow rate of 1 L/h [15,16]. For freeze-drying, the supernatant solutions were frozen at −80°C for 12 h and then freeze-dried for 24 h. All samples were collected and stored at 4°C until further analysis.

### 2.3 Cell culture and MTT assay

The experimental procedure outlined in the manuscript adheres to a standardized approach, incorporating specific adaptations from a previously established protocol [12] for evaluating cell culture and cytotoxicity. The RAW 264.7 cells, which are murine macrophages, were cultured in Dulbecco’s Modified Eagle Medium. The cells underwent different treatments, including exposure to LPS at a fixed concentration of 1 μg/mL and varying concentrations (ranging from 25 to 1,000 μg/mL) of R-COS for 24 h at a constant temperature of 37°C. After the incubation period, the evaluation of cell viability in the culture medium was performed using the MTT (3-(4,5-dimethylthiazol-2-yl)-2,5-diphenyltetrazolium bromide) assay, a widely used method to assess cellular metabolic activity and viability. Afterward, the absorbance at 540 nm was measured using a UV spectrophotometer for each well containing treated cells. The absorbance measurement is directly related to the quantity of formazan dye generated, and it functions as a reliable indicator of cell viability. The experiments were conducted in triplicate to ensure the reliability and reproducibility of the results. The data collected are presented as mean values ± standard error (SE), which represents both the central tendency and variability of the observations obtained from these experimental replicates.

### 2.4 Determination of NO production

The quantification of NO production was performed using a methodology based on the one developed by Yang et al. [17], with several modifications. The RAW 264.7 cells were initially cultured in plates with a density of 1 × 10^5^ cells per milliliter and treated for 24 h with LPS at a dosage of 1 μg/mL to induce the production of NO. After the period of incubation, the amount of nitrite, a stable byproduct of NO oxidation, was measured in the culture medium to determine the level of NO production. A microplate reader was used to make a spectrophotometric measurement of absorbance at 540 nm in order to determine the absorbance of the resultant solution. In each experiment, a new culture medium was utilized as a blank to account for any absorbance from the background. The nitrite content was measured by comparing it to a calibration curve prepared using a sodium nitrite standard. The experiments were performed three times to ensure reproducibility, and the data were analyzed and presented as mean values ± SE to illustrate the consistency and reliability of the results.

### 2.5 TNF-α, IL-6, IL-1β, and prostaglandin E2 (PGE2) measurement by enzyme-linked immunosorbent assay (ELISA)

Prior to treatment, the samples were dissolved using dimethyl sulfoxide and subsequently diluted with PBS in order to quantify the generation of pro-inflammatory cytokines (TNF-α, IL-1β, and IL-6) and PGE2. The assessment of the inhibitory effect of these samples on the production of proinflammatory cytokines and PGE2 from LPS-stimulated RAW 264.7 cells was performed using ELISA kits according to the manufacturer’s instructions. Each experiment was conducted thrice to ensure accuracy and reliability. The ELISA kits were used to quantify the concentrations of cytokines (IL-1β, TNF-α, and IL-6) and PGE2 in the cell culture supernatants. The collected data were subsequently analyzed and expressed as mean values ± SE to accurately depict the outcomes and the reliability of the experimental findings.

### 2.6 Determination of inflammatory factor levels

The ELISA technique was employed to quantify the amounts of inflammatory factors in RAW 264.7 cells stimulated with LPS and treated with R-COS. The cells were originally arranged in a 24-well plate with a density of 8 × 10^4^ cells per well and kept in a controlled environment for 24 h. Subsequently, the cells underwent pre-treatment with different concentrations (1, 5, 10, and 15 μg/mL) of COS for 1 h. Afterward, the cells were exposed to 500 ng/mL of LPS for an additional 24 h, while keeping the drug concentrations constant. Following the 24 h exposure period, the liquid above the sediment was meticulously gathered and then underwent centrifugation at a speed of 2,000 revolutions per minute and a temperature of 4°C for 5 min. The resulting liquid was subsequently preserved in a tube at a temperature of −20°C. The concentrations of different cytokines, such as TNF-α, IL-1β, IL-6, and IL-10, were measured using commercially available ELISA kits. In addition, the concentration of NO was measured using colorimetric detection kits. This method allowed for the assessment of the influence of R-COS on the levels of important inflammatory molecules in the liquid surrounding the cell culture. This provided valuable information about the potential anti-inflammatory properties of R-COS in conditions induced by LPS.

### 2.7 Measurement of calcitonin gene-related peptide (CGRP), serotonin (5-HT), endothelin (ET), and NO levels in zebrafish

To evaluate the anti-inflammatory effects in a live zebrafish model, a methodology based on the work of Vimalraj et al. [18,19] was utilized with minor adjustments. Zebrafish embryos were gathered and meticulously transferred using a pipette to 12-well plates, with an approximate quantity of 10–15 embryos per well, each containing 2 mL of embryo medium. The embryos were incubated for a duration of 7–9 h after fertilization (hpf). After an initial incubation period of 1 h, the embryo medium was enhanced with 5 μg/mL of LPS to provoke inflammation. The embryos were then incubated for an additional 15–17 h post fertilization (hpf) at a temperature of 28.5°C. After the period of inducing inflammation, the embryos were examined and photographed using a fluorescent microscope that had a Moticam color digital camera (Motix, Xiamen, China) attached to it. This procedure facilitated the observation and evaluation of cell death, ROS, and NO production in zebrafish embryos experiencing inflammatory conditions. The experiments were performed three times, and the obtained data were analyzed to determine the mean values along with the SE. This allowed for the measurement and comparison of the anti-inflammatory effects of the compounds or interventions being studied in this zebrafish model. In addition, the plasma levels of CGRP, 5-HT, and ET were assessed using the radioimmunoassay method, following the instructions provided by the manufacturer. We used specialized detection kits specifically designed for radioimmunoassay to accurately measure the levels of these substances in plasma samples. The procedure strictly followed the manufacturer’s protocols to ensure precise measurement of CGRP, 5-HT, and ET levels in the adult zebrafish plasma. This technique utilized the principles of radioimmunoassay to accurately measure the concentrations of these biomolecules, enabling meticulous analysis and comparison of their levels in the plasma samples.


**Ethical approval:** The animal experimental procedures conducted in this study comply with the standards established by the institutional ethical committee at Saveetha University, India (BRULAC/SDCH/SIMATS/IAEC/05-2021//123) and the Hospital of Nanjing Medical University, China (CHEC, 2023, 0015R).

### 2.8 Assessment of analgesic on AA-induced pain-like behavior in zebrafish model

The objective of the study was to confirm the occurrence of pain-related behaviors in zebrafish by utilizing specific behavioral parameters that were previously established in other zebrafish models [20,21]. We selected several distinct behavioral phenotypes that are relevant and specific to pain-related responses in zebrafish. The quantification of abdominal writhing-like behavior, which is a distinct response linked to visceral pain, was performed using a systematic methodology. Photographs of zebrafish in the sagittal plane were taken every 30 s, resulting in a total of 24 images per fish. Image analysis was performed utilizing the ImageJ 1.52 software designed for Windows operating system. The process entailed choosing three specific locations on the fish’s body: the frontal point (located at the front of the head), the central point (midway between the anal and dorsal fins), and the posterior point (positioned at the caudal fin). The fish’s body posture was evaluated by subtracting the measured angles from 180°, resulting in a quantifiable representation of the fish’s body curvature that indicates writhing-like behavior. In addition, the measurement of other pain-related behaviors involved evaluating the duration that the fish spent in the upper section of the experimental tank and analyzing circling behaviors. The duration spent in the upper region of the tank was determined using automated video-tracking software (Any-Maze™, Stoelting, CO, USA), which enabled precise measurement of the time spent in this specific area. In addition, the frequency of circling behaviors, a repetitive swimming pattern that suggests discomfort or abnormal motor behavior, was measured using the same video-tracking software. This software allowed for the monitoring of zebrafish movements at a speed of 30 frames per second, making it easier to precisely identify and measure circling behaviors. The choice of these particular behavioral parameters, namely abdominal writhing-like behavior, time spent in the top area of the tank, and circling behaviors, was determined based on their established significance in previous zebrafish pain models. These measures offer strong indicators of pain-related responses and act as dependable metrics to assess the effectiveness of interventions, such as R-COS, in reducing pain-associated behaviors in zebrafish models.

### 2.9 Real-time RT-PCR analysis

The RNA extraction process began by using TRIzol reagent (Invitrogen, Carlsbad, CA, USA) to isolate RNA from the samples. The mRNA levels were subsequently measured using the SYBR Green method, which utilized the StepOne Real-Time PCR System from Applied Biosystems in Carlsbad, CA, USA. The mRNA analysis utilized GAPDH, a frequently employed housekeeping gene, as the endogenous control for normalizing the gene expression data. The relative expression levels of the mRNAs were determined using the 2^–△△Ct^ method, which is a commonly used approach in quantitative real-time PCR (qPCR) analysis. This approach involves comparing the threshold cycle (*C*_t_) values of the target gene with the reference gene (GAPDH) to quantify the change in mRNA expression between different experimental conditions. The 2^–△△Ct^ method facilitates the evaluation of relative alterations in gene expression levels, enabling comparisons between different experimental groups or conditions (Table 1).

**Table 1:** List of primer sequences used for real-time RT-PCR study

Gene		5′→3′ Sequence
COX-2	Forward	5′-GCAAATCCTTGCTGTTCCAATC-3′
	Reverse	5′-GGAGAAGGCTTCCCAGCTTTTG-3′
iNOS	Forward	5′-ACAACGTGAAGAAAACCCCTTGTG-3′
	Reverse	5′-ACAGTTCCGAGCGTCAAAGACC-3′
GAPDH	Forward	5′-AGAAGGCTGGGGCTCATTTG-3′
	Reverse	5′-AGGGGCCATCCACAGTCTTC-3′
iNOS	Forward	5′-GGA GAT GCA AGG TCA GCT TC-3′
	Reverse	5′-GGC AAA GCT CAG TGA CTT CC-3′
COX-2a	Forward	5′-CCT GTT GTC AAG GTC CCA TT-3′
	Reverse	5′-TCA GGG ATG AAC TGC TTC CT-3′
β-actin	Forward	5′-CGA GCG TGG CTA CAG CTT CA-3′
	Reverse	5′-GAC CGT CAG GCA GCT CAT AG-3′

### 2.10 Statistical analysis

The study consisted of three individual and independent experimental trials carried out to ensure the reliability and strength of the obtained results. The data obtained from these experiments were subsequently gathered and displayed as the mean value, accompanied by the standard deviation (SD), which represents the extent of variation or diversity within the dataset. By incorporating the SD, one can gain a clear comprehension of the dispersion of data points around the mean, which offers valuable insights into the reliability and consistency of the data. To ascertain the statistical significance of the disparities among the different groups or conditions investigated in the study, a one-way analysis of variance (ANOVA) was employed. ANOVA is a statistical method used to determine if there are significant differences in the means of three or more groups. Tukey’s *post hoc* test, a statistical technique employed for pairwise comparisons, was used after doing the ANOVA analysis to identify significant differences across the various groups. Tukey’s test is a valuable technique for detecting significant disparities among groups, enabling us to ascertain whether circumstances or treatments had different and noteworthy impacts.

## 3 Results and discussion

### 3.1 Effect of R-COS or LPS on RAW264.7 macrophage cell viability

The LPS compound is renowned for its potent capacity to induce macrophages, resulting in the release of crucial pro-inflammatory cytokines such as IL-6 and TNF-α, as well as NO and the activation of NF-κB [22,23]. Significant scientific evidence has emphasized the strong correlation between the generation of ROS and inflammatory reactions [22,23]. To combat inflammation, researchers have developed a range of substances that have proven effective in suppressing these inflammatory responses [24,25]. R-COS shows great potential as a candidate for the anti-inflammatory process. However, the precise mechanism underlying the action of COS remains somewhat mysterious. The main goal of this study is to investigate the detailed mechanism by which R-COS, derived from CS, affects LPS-activated RAW264.6 macrophage cells. R-COS was synthesized as described in Section 2 [11] and used for the current study.

**Figure 1:**
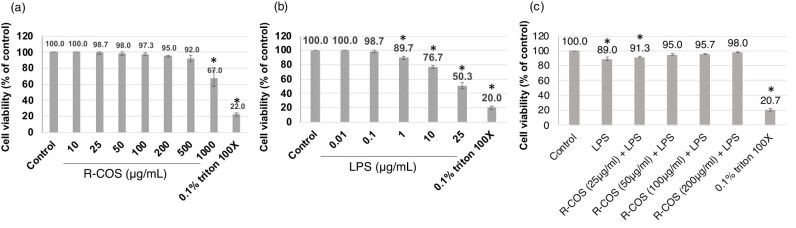
R-COS demonstrated a protective effect against LPS-induced cell death in RAW264.7 macrophage cells. (a) Cells were treated with different concentrations of R-COS (25–1,000 μg/mL) for 24 h. (b) Cells were treated with different concentrations of LPS (ranging from 0.001 to 10 μg/mL) for 24 h. (c) Cells were pre-treated with R-COS (25–500 μg/mL) for 24 h, followed by exposure to 1 μg/mL LPS for an additional 24 h. Cell viability was assessed using the MTT assay. Results are presented as mean ± SD, *n* = 3. * indicates statistical significance (*p* < 0.05) compared to control cells, while # indicates statistical significance (*p* < 0.05) compared to the LPS group.

This study investigated the protective efficacy of R-COS by inducing cytotoxicity in RAW264.7 cells through the use of LPS. The effect of different concentrations of R-COS on the survival of cells is illustrated in Figure 1a. Remarkably, the administration of R-COS at concentrations ranging from 25 to 500 μg/mL did not result in any significant harm to RAW264.7 cells when compared to the control group (which did not receive R-COS treatment) (*p* > 0.05). Nevertheless, when exposed to a higher concentration of R-COS (1,000 μg/mL), there was a significant decrease in cell viability (*p* < 0.05). Since the cell viability of COS-treated cells at concentrations ranging from 25 to 500 μg/mL was similar to that of the control group, these concentrations were chosen for further investigations. Moreover, when assessing the influence of various concentrations of LPS (ranging from 0.001 to 10 μg/mL) on cell viability (as shown in Figure 1b), it was noted that higher concentrations of LPS (1–10 μg/mL) significantly reduced cell viability compared to the control group (*p* < 0.05). This observation demonstrated the detrimental impact of increased LPS levels on the survival of cells, paving the way for further investigation into the potential safeguarding effects of R-COS against LPS-induced cell damage.

During a comparative analysis, it was observed that RAW264.7 cells treated with 10 μg/mL LPS showed a reduced cell viability of 75.08% in comparison to those treated with 1 μg/mL LPS, which had a cell viability of 84.73%. After comparing the results, the concentration of LPS at 1 μg/mL was selected for further trials because it had a lower cytotoxic effect. To examine the ability of R-COS to reduce LPS-induced cell damage, cells were first exposed to varying concentrations of R-COS (ranging from 25 to 500 μg/mL) for 24 h. Subsequently, the cells were then exposed to 1 μg/mL of LPS for an additional 24 h. Figure 1c shows that cells treated with 1 μg/mL LPS had lower viability than the untreated control group (*p* < 0.05).

Nevertheless, the application of higher concentrations of R-COS (100–500 μg/mL) before treatment effectively suppressed the cytotoxic effects induced by LPS (*p* < 0.05). The cells treated with R-COS at a concentration of 500 μg/mL exhibited the most significant inhibitory effect, indicating a strong protective influence. Our research indicates that R-COS, at concentrations ranging from 25 to 500 μg/mL, did not cause any harmful effects on RAW264.7 cells. However, when the concentration was increased to 1,000 μg/mL, it resulted in decreased cell viability. Although R-COS generally displayed scavenging properties against produced free radicals, excessively elevated levels likely increased intracellular oxidant levels, which contributed to the death of cells. These findings align with research conducted by Ei et al. [26], which documented a reduction in the viability of human EA.hy926 endothelial cells when exposed to COS levels surpassing 500 μg/mL. This underscores the importance of R-COS concentration, with 500 μg/mL being identified as the optimal level in this study. Moreover, the selection of LPS concentration had a substantial impact on cell viability, with the highest concentration employed being 1 μg/mL.

### 3.2 Anti-inflammatory properties of R-COS

**Figure 2:**
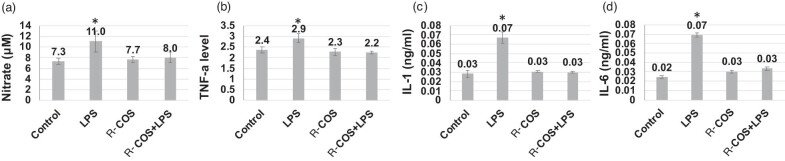
R-COS demonstrated a suppressive effect on pro-inflammatory substances, including cytokines TNF-α and IL-6, as well as NO, and NF-κB expression in LPS (1 μg/mL)-activated RAW264.7 cells. Cells were pre-treated with 500 μg/mL of R-COS for 24 h, followed by stimulation with 1 μg/mL of LPS for an additional 24 h. Levels of nitrite (a), TNF-α concentration (b), IL-1 amount (c), and IL-6 (d) were evaluated using an ELISA assay on the cultured media. Results are presented as mean ± SD from four independent experiments. * indicates statistical significance (*p* < 0.05) compared to control cells, and # indicates statistical significance (*p* < 0.05) compared to the LPS-stimulated group. R-COS significantly suppressed the expression of pro-inflammatory markers in RAW264.7 cells activated by LPS.

**Figure 3:**
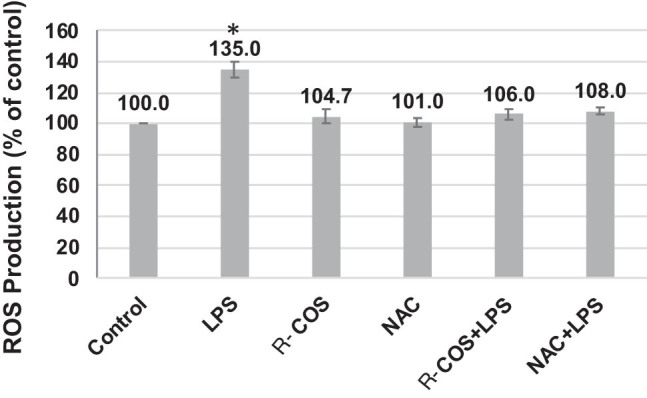
R-COS reduces intracellular ROS induced by LPS in RAW264.7 cells. RAW264.7 cells were treated with 1 μg/mL LPS for 24 h, with or without simultaneous treatment of 500 μg/mL R-COS. Additionally, cells were exposed to 1 μg/mL LPS for 6 h following a 1 h pre-treatment with R-COS (500 μg/mL). Intracellular ROS generation was assessed using DCFH-DA fluorescence assay. Data are presented as mean ± SD from three independent experiments (*n* = 3). Statistical significance was determined using a significance level of *p* < 0.05. * indicates statistical significance compared to the control group, and # indicates statistical significance compared to the LPS-stimulated group.

The experiment aimed to assess the anti-inflammatory effects of COS in RAW264.7 cells that were subjected to inflammation caused by LPS stimulation. Previous studies have shown that the presence of LPS in RAW264.7 cells initiates a cascade of inflammatory responses, resulting in elevated levels of IL-6, NO, and TNF-α proteins [27,28]. The study discovered that R-COS has the ability to diminish LPS-induced inflammation by lowering the levels of NO, TNF-α, and IL-6 proteins. These proteins are widely recognized as indicators of pro-inflammatory reactions triggered by LPS [6,7,12,15,29]. Figure 2a demonstrates that cells stimulated by LPS exhibited significantly elevated levels of NO in comparison to the control group (*p* < 0.05). However, when R-COS was administered before LPS activation, there was a notable decrease in NO levels. In addition, the levels of NO in cells that were stimulated with LPS and pre-treated with R-COS were shown to be similar to those in the control group (*p* > 0.05). This indicates that R-COS demonstrates potent anti-inflammatory characteristics. Similarly, the concentrations of TNF-α (Figure 2b) and IL-6 (Figure 3b) exhibited a consistent trend that correlated with the decrease in NO levels following R-COS pre-treatment. The observed reduction in cytokine levels following R-COS pre-treatment indicates an effective suppression of mediator release induced by LPS. Furthermore, the results were reinforced by real time RT-PCR analysis, which showed that R-COS effectively inhibited the expression of NF-κB in comparison to cells treated with LPS (*p* < 0.05) (Figure 2d). These findings are consistent with previous research that suggests that R-COS can reduce inflammation caused by LPS [27]. Macrophage cells are frequently used as a model to evaluate the effectiveness of bioactive compounds in combating inflammation. This is because these cells have a crucial role in producing pro-inflammatory substances when stimulated by LPS [30]. The decrease in NF-κB expression observed in cells pre-treated with R-COS before LPS exposure indicates that R-COS has anti-inflammatory properties against cells activated by LPS. The activation of the NF-κB signaling pathway leads to the generation of pro-inflammatory cytokines, including IL-6 and TNF-α [31]. Therefore, the decreased presence of NF-κB in cells exposed to R-COS suggests its ability to control inflammatory reactions in LPS-induced inflammation.

### 3.3 Impact of R-COS on the inhibition of ROS in LPS-activated RAW264.7 cells

The inquiry aimed to evaluate the impact of R-COS in inhibiting the generation of intracellular ROS triggered by LPS in RAW264.7 cells. Prior research has verified that LPS can significantly enhance the generation of ROS within cells. The study observed the capacity of R-COS to hinder intracellular ROS levels in cells exposed to LPS for different durations of incubation (ranging from 1 to 24 h), as depicted in Figure 3. The results revealed a significant increase in intracellular ROS levels in the group treated exclusively with LPS, particularly at the 3 h time point, compared to the control group (*p* < 0.05). In contrast, treatment with R-COS or NAC (a recognized ROS scavenger) in isolation did not result in any noteworthy change in ROS production.

Nevertheless, the prior exposure to R-COS and NAC significantly reduced the elevation of intracellular ROS levels caused by LPS. This suggests that R-COS exhibited the capacity to shield cells from the generation of ROS induced by LPS. ROS has a significant impact on the development of diseases when it accumulates excessively. The excessive creation of ROS often initiates inflammation by increasing the levels of pro-inflammatory cytokines, such as TNF-α and IL-6. By this notion, the current study noted elevated levels of TNF-α and IL-6 in response to ROS treatment. Nevertheless, when R-COS is present, the elevated levels of TNF-α and IL-6 that are triggered by LPS treatment are significantly decreased. This suggests that R-COS can reduce inflammation caused by ROS production.

### 3.4 R-COS inhibited the expressions of cyclooxygenase-2 (COX-2) and inducible nitric oxide synthase (iNOS) and the release of NO and PGE2

**Figure 4:**
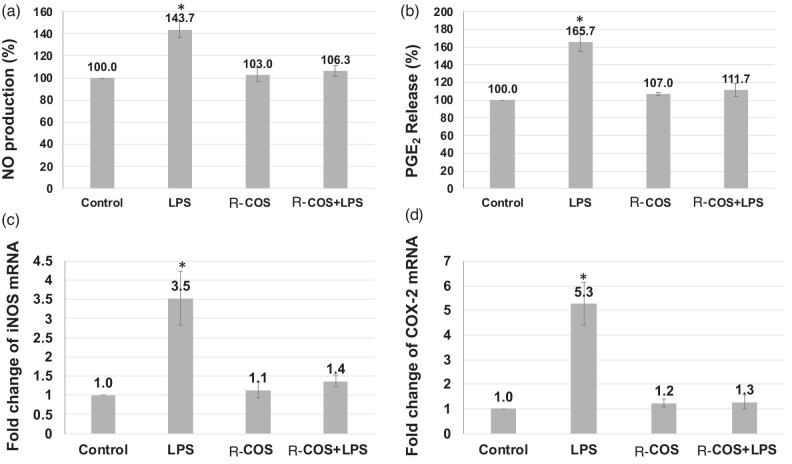
Suppressive impact of R-COS on the expression of NO (a) and PGE2 (b) iNOS (c) and COX-2 (d) mRNAs in LPS-stimulated RAW 264.7 cells. Data are presented as mean ± SD from three independent experiments (*n* = 3). The asterisks indicate notable variations in LPS-treated RAW 264.7 cells, where * denotes a significant difference at *p* < 0.05.

Figure 4a and b demonstrates that the chemical R-COS successfully suppressed the production of COX-2 and iNOS proteins that were stimulated by LPS. The R-COS therapy effectively suppressed the expression of iNOS. The levels of iNOS and COX-2 in cells that were activated with LPS were quantified. The amounts of these cells were markedly reduced when they were co-treated with R-COS. Furthermore, the concentrations of NO and PGE2 exhibited a considerable increase following LPS administration, as compared to the untreated control group. However, R-COS exhibited a notable decrease in the levels of NO and PGE2, which was directly influenced by the dosage given, as depicted in Figure 4c. The findings suggest that R-COS has the potential to reduce inflammation in LPS-stimulated RAW 264.7 cells by reducing the expression of COX-2, iNOS, and pro-inflammatory cytokines. This action is most likely accomplished by regulating the NF-κB and MAPK pathways.

### 3.5 R-COS reduced LPS-induced ROS and NO in zebrafish embryos

**Figure 5:**
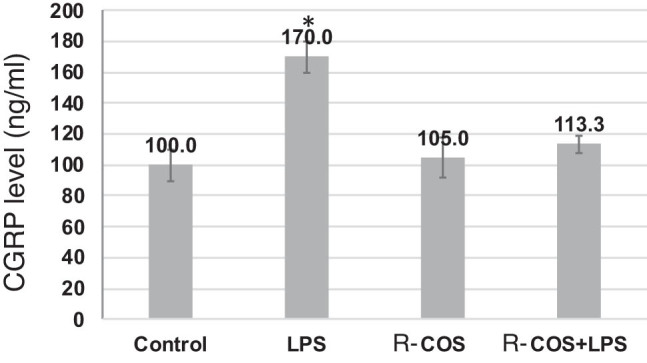
Effects of R-COS on CGRP in LPS-induced RAW macrophages. Data are presented as mean ± SD (*n* = 3). ^#^ *p* < 0.05 are significantly different compared with the control group.

The study examined how R-COS inhibits ROS and NO production in zebrafish embryos induced by LPS. This was done by using specific fluorescent probe dyes, namely acridine orange, DCFH-DA, and DAF-FM DA. Zebrafish embryos exhibited noticeable cellular harm when exposed to LPS, in contrast to the control group. Nevertheless, the administration of R-COS significantly reduced this harm, as shown in Figure 5a via fluorescence micrographs. Significantly, the fluorescence micrograph of zebrafish embryos treated with R-COS did not display the usual pattern observed in those treated with LPS. The analysis of ROS levels demonstrated a notable rise in zebrafish embryos treated with LPS, which was reduced when administered with R-COS (Figure 5b).

Similarly, the levels of NO in zebrafish embryos exposed to LPS were found to increase. However, treatment with R-COS resulted in a reduction (Figure 5c). The results indicate that R-COS successfully prevented the harmful effects of LPS on cell death, NO, and ROS levels in zebrafish embryos. This highlights the potential of R-COS as a protective agent against LPS-induced damage.

### 3.6 Effects of R-COS on neurogenic inflammation in LPS-stimulated RAW macrophages

The increase in CGRP expression observed after LPS stimulation is consistent with previous research indicating its correlation with inflammatory responses in macrophages (Figure 5). CGRP, a neuropeptide, is recognized for its role in intensifying neurogenic inflammation through the facilitation of vasodilation and the stimulation of the release of inflammatory mediators. The significant reduction in CGRP levels after R-COS administration suggests that it can regulate neurogenic inflammation in macrophages. The suppression observed in this case can be attributed to the anti-inflammatory properties of R-COS. R-COS may disrupt the signaling pathways responsible for CGRP expression or prevent the release of inflammatory factors induced by LPS. Furthermore, the decrease in CGRP expression indicates that R-COS may have a therapeutic function in alleviating conditions associated with neurogenic inflammation. This discovery emphasizes the significance of investigating R-COS as a potential option for controlling inflammatory reactions, especially those influenced by neurogenic factors. However, additional investigations are necessary to clarify the exact molecular mechanisms by which R-COS influences the expression of CGRP and neurogenic inflammation. Furthermore, it is imperative to conduct *in vivo* studies and clinical trials to authenticate the effectiveness and safety of R-COS as a therapeutic agent for neurogenic inflammatory disorders. To summarize, the current study shows that administering R-COS effectively reduces the expression of CGRP in RAW macrophages stimulated by LPS. This suggests that R-COS has the potential to be a valuable agent in the treatment of neurogenic inflammation. Additional investigation in this field may reveal new therapeutic approaches for addressing inflammatory conditions linked to neurogenic factors.

### 3.7 R-COS reduced COX-2 and iNOS in LPS-stimulated zebrafish embryos

**Figure 6:**
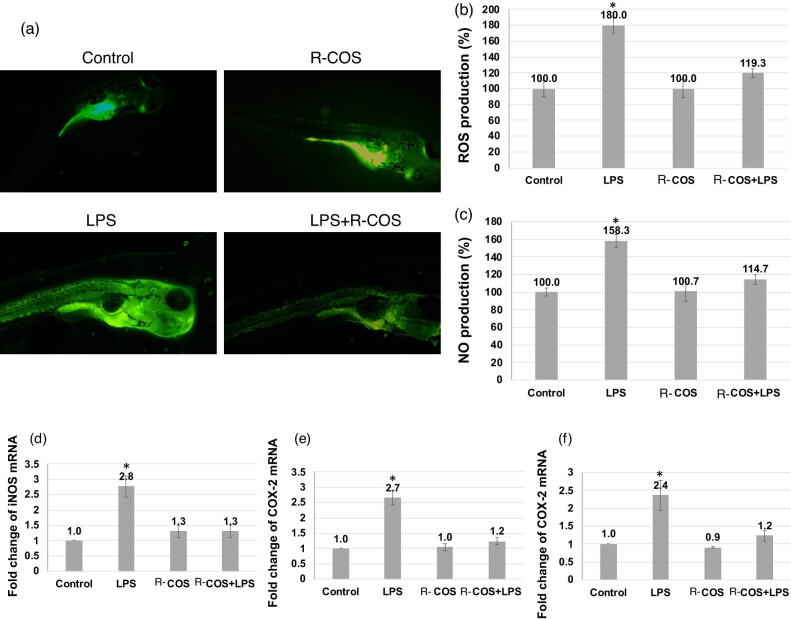
The study evaluated the suppressive effects of R-COS on multiple factors in LPS-induced zebrafish embryos, focusing on apoptosis (a) and (b), ROS production (c), NO production (d), iNOS miRNA expression (e), and COX-2a mRNA expression (f). ROS and NO levels were measured using DCF-DA and DAF-FM-DA stains, respectively. Pro-inflammatory cytokine expression was assessed, with fluorescence micrographs illustrating these evaluations shown above and quantified using ImageJ analysis below. Each experiment was conducted in triplicate using 15 larvae per group. Statistical significance (*p < 0.05) was determined. This comprehensive analysis provides insights into the potential inhibitory effects of R-COS on inflammation and cellular responses in LPS-induced zebrafish embryos.

The effects of R-COS on zebrafish with LPS-induced inflammation were assessed, showing significant suppression of COX-2 and iNOS protein levels, as depicted in Figure 6. Notably, the injection of R-COS led to a substantial reduction in the expression of COX-2. As a result, there was a significant decrease in the levels of iNOS and COX-2 proteins in zebrafish embryos that were activated with LPS and treated with R-COS. This is consistent with previous laboratory experiments conducted outside of a living organism, which reinforces the strong ability of R-COS to inhibit the production of proteins that promotes inflammation. The zebrafish, as an animal model, is important in a range of studies, especially those focused on diseases related to inflammation. The zebrafish model in this study exhibited the stimulation of ROS and NO generation by administering LPS, resulting in cellular demise.

Nevertheless, the administration of R-COS treatment significantly alleviated these effects without exhibiting any toxic properties. Furthermore, it was noted that the administration of LPS at a concentration of 5 μg/mL resulted in the manifestation of toxic effects such as yolk sac edema and an elevated heart rate. Conversely, the application of LPS at a concentration of 1 μg/mL caused a moderate level of cell death in zebrafish embryos. Surprisingly, the group of zebrafish embryos treated with R-COS did not show any harmful effects, indicating that R-COS can reduce the inflammatory responses caused by LPS. Furthermore, the R-COS treatment exhibited a strong inhibition of iNOS and COX-2 expressions in zebrafish stimulated with LPS, providing additional evidence for its anti-inflammatory characteristics.

### 3.8 Effect of R-COS on pain-like behaviors in zebrafish

**Figure 7:**
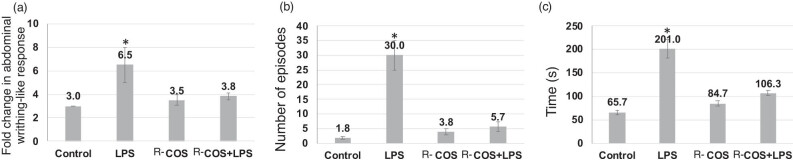
Primary pain-related behaviors exhibited by adult zebrafish following intraperitoneal injection of AA, with and without R-COS. Each experiment was performed in triplicate with ten adult zebrafish per group. Statistical significance (*p < 0.05) was determined.

To verify the occurrence of pain-like reactions in zebrafish, the impact of AA, both with and without R-COS, was assessed in the experimental group (Figure 7). As anticipated, AA induced a reaction resembling writhing by causing a five-fold increase in belly curvature compared to PBS. Furthermore, fish that were administered AA exhibited a predictable circling behavior and allocated a greater amount of time toward the upper region. While R-COS co-administration prevented these behaviors, for writhing-like response, circling behavior, and time spent in the top, respectively. In the investigation of R-COS effects on pain-like behaviors in zebrafish, AA was used to induce pain responses in the absence or presence of R-COS (Figure 7). Zebrafish exposed to AA exhibited a notable writhing-like response characterized by a significant increase in abdominal curvature, approximately five times higher compared to those administered with PBS.

Additionally, fish injected with AA displayed stereotypic circling behavior and spent an increased amount of time at the top of the experimental tank. However, when R-COS was co-administered with AA, a mitigation of these pain-like behaviors was observed. Zebrafish subjected to R-COS co-treatment displayed reduced writhing-like responses, decreased circling behavior, and a lesser duration spent at the top of the tank compared to those solely exposed to AA. The induction of pain-like responses in zebrafish through AA administration validated the experimental model used in this study. The substantial increase in abdominal curvature observed in response to AA aligns with previous studies, confirming its efficacy in inducing visceral pain-like behaviors in zebrafish [20,21]. Moreover, the observed circling behavior and increased time spent in the top of the tank further substantiate the manifestation of pain-like responses induced by AA.

The significant reduction in pain-like behaviors, including writhing responses, circling behavior, and altered swimming patterns in the presence of R-R-COS, suggests its potential analgesic effects in mitigating nociceptive responses in zebrafish. The ability of R-COS to ameliorate these pain-associated behaviors hints at its role in modulating nociceptive signaling pathways or its potential to interfere with pain transmission mechanisms. These findings highlight the promising analgesic properties of R-COS and suggest its potential as a therapeutic agent in managing pain-related conditions. However, further investigations are necessary to elucidate the precise mechanisms underlying the analgesic effects of R-COS in zebrafish models. Additionally, studies assessing the long-term effects and safety profile of R-COS administration in alleviating pain-like behaviors would be beneficial to ascertain its viability as a potential analgesic agent. The results indicate that R-COS co-administration effectively attenuates pain-like responses induced by AA in zebrafish. This signifies the potential of R-COS as a candidate for further exploration in the development of novel analgesic interventions for pain management.

## 4 Conclusion

This study thoroughly examined the anti-inflammatory and analgesic characteristics of R-COS obtained from CS, both in laboratory settings and in living organisms. R-COS conducted a sequence of experiments that showed notable anti-inflammatory effects in RAW 264.7 cells, a widely utilized macrophage model, without causing any harm to the cells. In laboratory tests, it was found that R-COS significantly decreased the production of substances that cause inflammation, such as NO, PGE2, iNOS, COX-2, and pro-inflammatory cytokines (TNF-α, IL-6, and IL-1), in LPS-stimulated RAW 264.7 cells. This suggests that R-COS has the potential to be used as an anti-inflammatory agent. Moreover, *in vivo* experiments performed on zebrafish embryos stimulated with LPS confirmed the effectiveness of R-COS in reducing inflammation. The researchers noted that R-COS provided protection to the zebrafish embryos against inflammatory stress and inhibited the production of important pro-inflammatory proteins, such as COX-2 and iNOS. Furthermore, R-COS demonstrated the capacity to regulate the levels of CGRP and NO, indicating its impact on neurogenic inflammation mechanisms. Furthermore, the study showcased the pain-relieving effects of R-COS in zebrafish models by reducing pain-related behaviors caused by AA. This discovery enhances the potential therapeutic use of R-COS in the management of pain-related conditions. Overall, the findings emphasize the encouraging prospects of CS-derived R-COS as a versatile therapeutic agent. The proven anti-inflammatory properties of R-COS, along with its capacity to relieve pain-related behaviors, make it a highly promising candidate for various applications in healthcare and tissue engineering. These findings emphasize the significance of conducting additional research and enhancing R-COS-based formulations for potential use in anti-inflammatory therapies and pain management strategies.
